# Supplementary material for: Altered microRNA Transcriptome in Cultured Human Liver Cells upon Infection with Ebola Virus
Source: Int J Mol Sci. 2021 Apr 6;22(7):3792. doi: 10.3390/ijms22073792 (PMC8038836; doi:10.3390/ijms22073792)
Supplement: Supplementary file 1 [file ijms-22-03792-s001.zip › Supplementary_File/C_ GO_Analysis_Results/16-30nt_go_Makona-96h-Huh7_vs_Control-96h-Huh7_up.mature_mirna_targets/CC_result(Human).html]

| GO.ID | Term | Ontology | Count | Pop.Hits | List.Total | Pop.Total | Fold.Enrichment | Pvalue | FDR | Enrichment.Score | Gene.Ratio | GENES |
| --- | --- | --- | --- | --- | --- | --- | --- | --- | --- | --- | --- | --- |
| GO:0098797 | plasma membrane protein complex | Cellular component | 13 | 574 | 152 | 18698 | 2.7860122868146 | 0.000838255839369204 | 0.720061766018146 | 3.0766234125523 | 0.0855263157894737 | RGS7//CACNA2D4//GJB2//KCNC4//KCNS1//STX1A//CDH24//SYNJ1//FAS//FKBP1A//HLA-DPB1//TFRC//SHISA6// |
| GO:0034703 | cation channel complex | Cellular component | 7 | 217 | 152 | 18698 | 3.96816638370119 | 0.00201278861523173 | 0.840812775908779 | 2.69620183269151 | 0.0460526315789474 | SHISA6//TRPC5//CACNA2D4//KCNC4//KCNS1//STX1A//FKBP1A// |
| GO:0098796 | membrane protein complex | Cellular component | 19 | 1166 | 152 | 18698 | 2.0045025728988 | 0.00293648233728328 | 0.840812775908779 | 2.53217260706127 | 0.125 | BAK1//SYNJ1//STX1A//CLTCL1//ATP6V0E1//RGS7//CACNA2D4//GJB2//KCNC4//KCNS1//CDH24//FAS//COX4I1//TMEM199//FKBP1A//SHISA6//TRPC5//HLA-DPB1//TFRC// |
| GO:0098552 | side of membrane | Cellular component | 10 | 509 | 152 | 18698 | 2.416761451763 | 0.00881349209107379 | 0.910004130498878 | 2.05485198087395 | 0.0657894736842105 | CD274//CD209//GFRAL//MCAM//TFRC//THBS1//FKBP1A//PTPN7//HLA-DPB1//RGS7// |
| GO:0034702 | ion channel complex | Cellular component | 7 | 296 | 152 | 18698 | 2.90909495021337 | 0.0107189717990316 | 0.910004130498878 | 1.96984687167899 | 0.0460526315789474 | SHISA6//TRPC5//CACNA2D4//KCNC4//KCNS1//STX1A//FKBP1A// |
| GO:0030424 | axon | Cellular component | 10 | 538 | 152 | 18698 | 2.28648992369399 | 0.0126468381415375 | 0.910004130498878 | 1.8980180396521 | 0.0657894736842105 | TIMP2//TRPC5//SYNJ1//FKBP1A//KCNC4//KIF1B//FAM168B//HEPACAM//STX1A//MUL1// |
| GO:0009986 | cell surface | Cellular component | 13 | 793 | 152 | 18698 | 2.01660914581536 | 0.012765263745678 | 0.910004130498878 | 1.89397020772152 | 0.0855263157894737 | CD274//CD209//GFRAL//MCAM//TFRC//THBS1//HYAL4//HLA-DPB1//FAS//LYZL6//CDH24//TIMP2//TNS1// |
| GO:0032991 | protein-containing complex | Cellular component | 54 | 5071 | 152 | 18698 | 1.30994094386034 | 0.0138393418457174 | 0.910004130498878 | 1.85888456302736 | 0.355263157894737 | RASSF2//WDR1//COL23A1//THBS1//PURB//NR6A1//HOXA9//ALX4//KIF1B//CAMK1G//AFF3//SPC24//WDHD1//GFRAL//DZIP1//PIP5K1A//CDK12//H2AFY2//BAK1//SYNJ1//STX1A//CLTCL1//MCCC2//GCLM//MAPKBP1//TMEM199//LARP4B//RARB//ATP6V0E1//ZC3H12A//TFRC//TAF11//COX4I1//RGS7//CACNA2D4//GJB2//KCNC4//KCNS1//CDH24//FAS//HLA-DPB1//FKBP1A//CUL2//RNF4//SHISA6//KAT7//TRPC5//PRKAA2//F2RL2//SLC41A1//MITF//NPPC//PGLYRP4//NCOA1// |
| GO:0031307 | integral component of mitochondrial outer membrane | Cellular component | 2 | 23 | 152 | 18698 | 10.6967963386728 | 0.0148516331879794 | 0.910004130498878 | 1.82822578570522 | 0.0131578947368421 | BAK1//MUL1// |
| GO:0034704 | calcium channel complex | Cellular component | 3 | 65 | 152 | 18698 | 5.67753036437247 | 0.0159321021917552 | 0.910004130498878 | 1.79772691660033 | 0.0197368421052632 | CACNA2D4//FKBP1A//TRPC5// |
| GO:1902495 | transmembrane transporter complex | Cellular component | 7 | 321 | 152 | 18698 | 2.68252992293819 | 0.0161092292891238 | 0.910004130498878 | 1.7929252369543 | 0.0460526315789474 | SHISA6//TRPC5//CACNA2D4//KCNC4//KCNS1//STX1A//FKBP1A// |
| GO:0031306 | intrinsic component of mitochondrial outer membrane | Cellular component | 2 | 24 | 152 | 18698 | 10.2510964912281 | 0.0161162882532823 | 0.910004130498878 | 1.79273497349448 | 0.0131578947368421 | BAK1//MUL1// |
| GO:0044464 | cell part | Cellular component | 145 | 16886 | 152 | 18698 | 1.05631338947867 | 0.0163571703386221 | 0.910004130498878 | 1.78629182368496 | 0.953947368421053 | RIPK3//FAM168B//DGKA//F2RL2//PPP1R13B//SLC41A1//GJB2//SIRPB2//CD274//CD209//HLA-DPB1//FAS//KCNC4//KCNS1//GFRAL//LTA//MCAM//MTM1//PMP22//DLL4//ACPP//TMEM100//CAMK1G//RGS7//HIF3A//CDH24//SMO//ST14//STX1A//TFRC//TRPC3//TRPC5//SLC30A2//PRRT1//PIP5K1A//NCOA1//VNN1//COL23A1//CACNA2D4//TRAF4//RASSF2//MFAP3L//WDR1//MOB3A//PGLYRP4//PYROXD2//CLTCL1//HYAL4//LYZL6//THBS1//TIMP2//TNS1//TMEM199//TACC1//TCHH//PDE1B//MUL1//CLEC4M//NOD1//WDHD1//LIX1L//MAP3K8//RUNDC3B//DHCR24//HEPACAM//FKBP1A//DZIP1//MAPKBP1//NBPF14//HOXA9//MITF//PCK1//NMD3//YBX2//QRICH1//PRKAA2//PTPN7//RARB//PBOV1//RNF4//NECAB3//PRDX2//ZNF185//ZC3H12A//C3ORF20//NBPF3//FUBP3//IDI2//NR6A1//ALX4//PIP5K1C//PRSS22//SHISA6//KIF1B//SYNJ1//CUL2//CDK12//COX4I1//SLC25A35//EXOG//LAPTM5//KAT7//TMEM170A//BAK1//H2AFY2//MCCC2//NCOA4//FOXP4//ZNF488//ZNF597//SPC24//ZNF320//CUX2//EBF3//ALX3//AFF3//NOVA2//PAX7//DBR1//PURB//ZNF329//SLTM//CDK2AP1//C2ORF16//ZNF251//CREB5//USP51//LARP4B//SUMF2//TAF11//LZTR1//GCLM//KRT6C//SATL1//ETNK2//KRTAP4-4//H6PD//MOGAT3//AGPAT4//SPAG17//KDM2A//ATP6V0E1//NPPC//COX18//KRTAP4-12// |
| GO:0005905 | clathrin-coated pit | Cellular component | 3 | 67 | 152 | 18698 | 5.50805184603299 | 0.0172697817562997 | 0.910004130498878 | 1.76271315071474 | 0.0197368421052632 | SYNJ1//TFRC//CLTCL1// |
| GO:1990351 | transporter complex | Cellular component | 7 | 329 | 152 | 18698 | 2.61730123180291 | 0.0181809156595416 | 0.910004130498878 | 1.74038424785376 | 0.0460526315789474 | SHISA6//TRPC5//CACNA2D4//KCNC4//KCNS1//STX1A//FKBP1A// |
| GO:0033176 | proton-transporting V-type ATPase complex | Cellular component | 2 | 26 | 152 | 18698 | 9.46255060728745 | 0.0187779579001872 | 0.910004130498878 | 1.726351638955 | 0.0131578947368421 | TMEM199//ATP6V0E1// |
| GO:0005623 | cell | Cellular component | 145 | 16915 | 152 | 18698 | 1.0545023881015 | 0.0189583286695702 | 0.910004130498878 | 1.72219995189125 | 0.953947368421053 | RIPK3//FAM168B//DGKA//F2RL2//PPP1R13B//SLC41A1//GJB2//SIRPB2//CD274//CD209//HLA-DPB1//FAS//KCNC4//KCNS1//GFRAL//LTA//MCAM//MTM1//PMP22//DLL4//ACPP//TMEM100//CAMK1G//RGS7//HIF3A//CDH24//SMO//ST14//STX1A//TFRC//TRPC3//TRPC5//SLC30A2//PRRT1//PIP5K1A//NCOA1//VNN1//COL23A1//CACNA2D4//TRAF4//RASSF2//MFAP3L//WDR1//KAT7//NECAB3//DHCR24//CUX2//MOB3A//PGLYRP4//PYROXD2//WDHD1//SPC24//H2AFY2//ZC3H12A//TCHH//YBX2//CDK12//KRT6C//PIP5K1C//MCCC2//TMEM199//LAPTM5//COX4I1//KIF1B//PCK1//BAK1//MUL1//NCOA4//EXOG//NOD1//MAP3K8//FKBP1A//DZIP1//LARP4B//GCLM//SATL1//AFF3//PDE1B//ETNK2//PRKAA2//PTPN7//PRDX2//CDK2AP1//CLTCL1//CUL2//KRTAP4-4//SYNJ1//IDI2//PRSS22//CLEC4M//FOXP4//ZNF488//ZNF597//ZNF320//EBF3//ALX3//NR6A1//HOXA9//MITF//NOVA2//PAX7//NMD3//DBR1//PURB//RARB//PBOV1//RNF4//ALX4//TACC1//ZNF329//SLTM//C2ORF16//FUBP3//ZNF251//CREB5//TMEM170A//LIX1L//RUNDC3B//HEPACAM//MAPKBP1//NBPF14//QRICH1//TNS1//ZNF185//C3ORF20//NBPF3//KDM2A//TAF11//USP51//SLC25A35//SUMF2//THBS1//H6PD//MOGAT3//AGPAT4//LZTR1//NPPC//SPAG17//HYAL4//LYZL6//TIMP2//ATP6V0E1//SHISA6//COX18//KRTAP4-12// |
| GO:0032592 | integral component of mitochondrial membrane | Cellular component | 3 | 70 | 152 | 18698 | 5.27199248120301 | 0.0193906296126561 | 0.910004130498878 | 1.71240808917488 | 0.0197368421052632 | COX18//BAK1//MUL1// |
| GO:0098573 | intrinsic component of mitochondrial membrane | Cellular component | 3 | 71 | 152 | 18698 | 5.1977390659748 | 0.020128147240371 | 0.910004130498878 | 1.6961961992949 | 0.0197368421052632 | COX18//BAK1//MUL1// |
| GO:0009897 | external side of plasma membrane | Cellular component | 6 | 287 | 152 | 18698 | 2.57170364936732 | 0.0300471595960869 | 1 | 1.52219657624193 | 0.0394736842105263 | CD274//CD209//GFRAL//MCAM//TFRC//THBS1// |
| GO:0019898 | extrinsic component of membrane | Cellular component | 6 | 289 | 152 | 18698 | 2.55390639227827 | 0.0309337098158787 | 1 | 1.50956799283387 | 0.0394736842105263 | PRSS22//ST14//FKBP1A//ZC3H12A//CDH24//RGS7// |
| GO:0031312 | extrinsic component of organelle membrane | Cellular component | 2 | 37 | 152 | 18698 | 6.64935988620199 | 0.036316014206846 | 1 | 1.43990182267741 | 0.0131578947368421 | ZC3H12A//FKBP1A// |
| GO:0032590 | dendrite membrane | Cellular component | 2 | 38 | 152 | 18698 | 6.47437673130194 | 0.0381331721637487 | 1 | 1.41869706580136 | 0.0131578947368421 | SHISA6//KCNC4// |
| GO:0008076 | voltage-gated potassium channel complex | Cellular component | 3 | 92 | 152 | 18698 | 4.01129862700229 | 0.0391404027205177 | 1 | 1.40737471014458 | 0.0197368421052632 | KCNC4//KCNS1//STX1A// |
| GO:0005886 | plasma membrane | Cellular component | 55 | 5503 | 152 | 18698 | 1.22946096387616 | 0.042383270691555 | 1 | 1.37280553208758 | 0.361842105263158 | TCHH//LYZL6//RGS7//PIP5K1C//HLA-DPB1//PRSS22//CLEC4M//F2RL2//FAS//ST14//TFRC//TRPC3//TRPC5//LAPTM5//CACNA2D4//SMO//GJB2//KCNC4//KCNS1//STX1A//CD274//CD209//GFRAL//MCAM//THBS1//PTPN7//NOD1//SLC41A1//CDH24//SYNJ1//SHISA6//PIP5K1A//FKBP1A//RIPK3//FAM168B//DGKA//PPP1R13B//SIRPB2//LTA//MTM1//PMP22//DLL4//ACPP//TMEM100//CAMK1G//HIF3A//SLC30A2//PRRT1//NCOA1//VNN1//COL23A1//TRAF4//RASSF2//MFAP3L//WDR1// |
| GO:0045095 | keratin filament | Cellular component | 3 | 97 | 152 | 18698 | 3.80453065653825 | 0.0446436769251695 | 1 | 1.35024004340405 | 0.0197368421052632 | KRT6C//KRTAP4-12//KRTAP4-4// |
| GO:0043596 | nuclear replication fork | Cellular component | 2 | 42 | 152 | 18698 | 5.8577694235589 | 0.0457362818603334 | 1 | 1.33973914434612 | 0.0131578947368421 | WDHD1//PURB// |
| GO:0031256 | leading edge membrane | Cellular component | 4 | 165 | 152 | 18698 | 2.98213716108453 | 0.0458478667155782 | 1 | 1.3386808670868 | 0.0263157894736842 | PIP5K1C//PIP5K1A//KCNC4//SHISA6// |
| GO:0034705 | potassium channel complex | Cellular component | 3 | 99 | 152 | 18698 | 3.72767145135566 | 0.0469472233825334 | 1 | 1.32839008828185 | 0.0197368421052632 | KCNC4//KCNS1//STX1A// |
| GO:0005891 | voltage-gated calcium channel complex | Cellular component | 2 | 43 | 152 | 18698 | 5.72154222766218 | 0.0477174085862968 | 1 | 1.32132314981374 | 0.0131578947368421 | FKBP1A//CACNA2D4// |
| GO:0005622 | intracellular | Cellular component | 128 | 14673 | 152 | 18698 | 1.07310599131236 | 0.0479822149402889 | 1 | 1.31891970813679 | 0.842105263157895 | KAT7//NECAB3//DHCR24//CUX2//HLA-DPB1//CAMK1G//RGS7//WDHD1//RASSF2//SPC24//H2AFY2//NCOA1//ZC3H12A//YBX2//CDK12//KRT6C//WDR1//MCCC2//TMEM199//LAPTM5//RIPK3//COX4I1//KIF1B//PPP1R13B//FAS//PCK1//BAK1//HIF3A//MUL1//NCOA4//EXOG//NOD1//MAP3K8//DGKA//FKBP1A//DZIP1//LARP4B//PIP5K1C//GJB2//GCLM//SATL1//AFF3//MTM1//PDE1B//ETNK2//PRKAA2//PTPN7//STX1A//PRDX2//TCHH//CDK2AP1//CLTCL1//PIP5K1A//CUL2//KRTAP4-4//SYNJ1//IDI2//FOXP4//ZNF488//ZNF597//ZNF320//EBF3//ALX3//NR6A1//HOXA9//MCAM//MITF//NOVA2//PAX7//NMD3//DBR1//ACPP//PURB//RARB//PBOV1//RNF4//ALX4//TACC1//ZNF329//SLTM//C2ORF16//FUBP3//ZNF251//CREB5//TRAF4//MFAP3L//TMEM170A//CLEC4M//LIX1L//RUNDC3B//HEPACAM//MAPKBP1//NBPF14//CD209//QRICH1//TNS1//TRPC5//ZNF185//SLC30A2//C3ORF20//NBPF3//KDM2A//TAF11//USP51//SLC25A35//TFRC//SUMF2//TMEM100//THBS1//COL23A1//H6PD//MOGAT3//AGPAT4//SMO//LZTR1//NPPC//SPAG17//ATP6V0E1//COX18//VNN1//TIMP2//KRTAP4-12//FAM168B//KCNS1//MOB3A//GFRAL//PGLYRP4//PYROXD2// |
| GO:0044459 | plasma membrane part | Cellular component | 31 | 2830 | 152 | 18698 | 1.3474939557374 | 0.0483229862844324 | 1 | 1.31584623484273 | 0.203947368421053 | PIP5K1C//CD274//CD209//GFRAL//MCAM//TFRC//THBS1//PTPN7//GJB2//PRSS22//ST14//CLEC4M//F2RL2//FAS//TRPC3//TRPC5//LAPTM5//PIP5K1A//KCNC4//NOD1//SLC41A1//SYNJ1//RGS7//CACNA2D4//KCNS1//STX1A//CDH24//FKBP1A//SHISA6//SMO//HLA-DPB1// |
